# *GRIA2/ENPP3* Regulates the Proliferation and Migration of Vascular Smooth Muscle Cells in the Restenosis Process Post-PTA in Lower Extremity Arteries

**DOI:** 10.3389/fphys.2021.712400

**Published:** 2021-08-24

**Authors:** Mi Zhou, Lixing Qi, Yongquan Gu

**Affiliations:** Department of Vascular Surgery, Xuanwu Hospital, Capital Medical University, Beijing, China

**Keywords:** restenosis, *GRIA2*, percutaneous transluminal angioplasty, peripheral artery disease, vascular smooth muscle cells

## Abstract

Restenosis is the main restriction on the long-term efficacy of percutaneous transluminal angioplasty (PTA) therapy for peripheral artery disease (PAD). Interventions to prevent restenosis are poor, and the exact mechanism is unclear. Here, we aimed to elucidate the role of *GRIA2* in the restenosis process post-PTA in lower extremity arteries. We searched the differentially expressed genes (DEGs) between atherosclerotic and restenotic artery plaques in the Gene Expression Omnibus (GEO), and five DEGs were identified. Combined with Gene Ontology (GO) enrichment analysis, *GRIA2* was significantly correlated with the restenosis process. Tissue samples were used to examine *GRIA2* expression by immunofluorescence staining of atherosclerotic and restenotic artery plaques. The regulation of *GRIA2* in vascular smooth muscle cells (VSMCs) was confirmed by lentiviral transfection. Overexpression of *GRIA2* promoted the proliferation and migration of VSMCs. Using Kyoto Encyclopedia of Genes and Genomes (KEGG) enrichment analysis and protein–protein interaction (PPI) network, a strong connection between *ENPP3* and *GRIA2* was discovered. *In vitro* results showed that the high expression of *GRIA2* in VSMCs enhanced the expression of *ENPP3*, while downregulation of *GRIA2* downregulated *ENPP3*. *GRIA2* is highly differentially expressed in restenotic arterial plaques, promoting the proliferation and migration of VSMCs through upregulation of *ENPP3*. These discoveries will help us to obtain a better understanding of restenosis in lower extremity arteries.

## Introduction

Peripheral artery disease (PAD) affects approximately 15–20% of persons over 70years of age (Criqui et al., 1985; Hiatt et al., 1995; Selvin and Erlinger, 2004), and chronic atherosclerotic ischemia of the lower extremities leads to a high amputation rate and mortality (Diehm et al., 2009; Zeller et al., 2009; Meves et al., 2010). Despite the wide use of percutaneous transluminal angioplasty (PTA), due to its minimal invasiveness and effectiveness (Apelqvist et al., 2011), restenosis occurs in up to 70% of patients within 1year (Liistro et al., 2013) and limits the long-term efficacy. Although there have been attempts to prevent or reduce restenosis pharmacologically through drug-coated balloons (DCBs; Schmidt et al., 2016; Peterson et al., 2017; Liistro et al., 2019), the restenosis rate is still high with almost 23% of patients developing it within 12months (Liistro et al., 2019). Restenosis has become the main obstacle to long-term efficacy post-PTA in lower extremity arteries. Elucidating the regulatory mechanism of restenosis post-PTA will aid in significantly improving PTA prognosis and the long-term patency rate of lower extremity arteries.

Excessive proliferation and migration of vascular smooth muscle cells (VSMCs) are the predominant pathogenic mechanisms of the restenosis process (Bennett et al., 2016). Usually, mature VSMCs maintain a contractile-like resting state and stay in the media of the vessels. PTA damages the vascular intima and causes physical injury thereby inducing the conversion of VSMCs to a proliferative phenotype followed by migration to the intima and proliferation, leading to restenosis (Newby and Zaltsman, 2000; Louis and Zahradka, 2010; Gomez and Owens, 2012). Despite decades of study, a sufficient understanding of restenosis pathogenesis remains elusive, which is why developing targeted therapeutic strategies has been difficult. Clarifying the mechanism of VSMCs migration and proliferation in restenosis may provide new research insights into the causes of restenosis, which may lead to treatment strategies that ameliorate the overall efficacy of PTA treatment.

Recent advances have facilitated efforts to determine restenosis pathogenesis. Gene sequencing technology can verify molecular abnormalities correlated with pathophysiology and identify therapeutic targets for diverse diseases (Grunewald et al., 2015; LeBlanc and Marra, 2015). Searching the Gene Expression Omnibus (GEO) database for data related to the development and progression of restenosis has helped researchers to track restenosis pathogenesis (Rabbani et al., 2016). In our study, two datasets (GSE23314 and GSE53274) were chosen from the GEO database. The two datasets included 11 restenotic (RS) plaque tissues and 99 atherosclerotic (AS) plaque tissues. The gene expression profiling data from these tissues were reanalyzed, and differentially expressed genes (DEGs) were identified. In addition to analyzing Gene Ontology (GO) enrichment data, glutamate ionotropic receptor AMPA type subunit 2 (*GRIA2*), which is a glutamate receptor (Hansen et al., 2007), was found to be significantly correlated with restenosis pathogenesis. Moreover, using Kyoto Encyclopedia of Genes and Genomes (KEGG) enrichment analysis, ENPP3 was predicted to be the downstream target of *GRIA2*, participating in the proliferation and migration of VSMCs in restenosis.

## Materials and Methods

### Data Collection and Microarray Data

We searched the GEO database for publicly available studies using the following keywords: “atherosclerosis OR restenosis OR restenotic plaque OR atherosclerotic plaque” (study keyword), “Homo sapiens” (organism), and “Expression profiling by array” (study type). Databases were searched for all data reported before July 30, 2020. The inclusion criteria were as follows: (1) restenosis tissue samples and arteriosclerotic lower extremity artery tissue samples and (2) mRNA expression profiles. After systematic analysis, two mRNA gene expression profiles (GSE23314 and GSE53274) were reviewed further. The GSE23314 dataset included six restenotic tissue samples and 95 arteriosclerotic tissue samples. The GSE53274 dataset contained four arteriosclerotic tissue samples and five restenotic tissue samples. The detection platforms used for mRNA sequencing of GSE23314 and GSE53274 were GPL4372 and GPL570 (Affymetrix), respectively.

### DEGs Data Processing

To identify common DEGs in the datasets, we used GEO2R, which is an interactive web tool that compares two or more groups of samples under the same experimental conditions in a GEO dataset.^1^ All the DEGs were identified according to the inclusion criteria of *p*<0.05. Bioinformatics & Evolutionary Genomics, which is an online website tool, was used to plot Venn diagrams of these two datasets. Each circle represents a dataset, and the overlapping area represents the target DEGs.

### GO and KEGG Pathway Enrichment Analyses of DEGs

The functional and pathway enrichment of the proteins encoded by candidate genes were analyzed, and these genes were annotated using the DAVID database.^2^ GO enrichment analysis of the molecular functions of the DEGs was performed using the DAVID database. KEGG analysis of the DEGs was performed using the KOBAS online analysis database.^3^

### Protein–Protein Interaction Network Integration

Cytoscape is a software system that is commonly used to identify the interactions between known proteins and predicted proteins (Doncheva et al., 2019). Each node is a gene or protein, and the connections between nodes represent the interactions between these biological molecules, which can be used to identify interactions and pathway relationships between the proteins encoded by the DEGs in our research. The corresponding proteins in the central node may be core proteins or key candidate genes with important physiological regulatory functions.

### Animal Model

Male Sprague–Dawley (SD) rats (~120g) were obtained from the Beijing Huafukang Bioscience Co., Inc. in China. During the entire study, all rats had free access to water and food. The animal care and experiments were performed in accordance with those in previous publications (Zhou et al., 2015, 2016). The protocol was authorized by the Ethics Committee of Xuanwu Hospital affiliated with Capital Medical University. All rats underwent surgical procedures under anesthesia with sodium pentobarbital.

A high-fat diet was provided to all rats throughout the experiment. All eligible rats were assigned to two groups: restenosis and atherosclerosis groups. To emphasize restenotic plaque alternations, rats from the atherosclerosis group were chosen as the control. The surgical procedures for the establishment of restenosis (*n*=6) and atherosclerosis (*n*=6; control group) models were as follows: For the restenosis group, first, a 1.5-mm wire-guided balloon catheter (Medtronic, Inc., Minnesota, America) was inserted into the iliac artery through the femoral artery. Then, endothelial denudation was achieved by inflating balloon catheters at 8, 10, and 12 atmospheric pressure (atm). Approximately 4weeks after the first surgical procedure, PTA was further performed for the induction of restenotic plaques if the iliac artery developed severe atherosclerotic damage, which was confirmed by ultrasound examination. Then, a color Doppler ultrasonogram was used to examine the formation of the stenosed iliac arteries after PTA. For the atherosclerosis group, rats received a sham surgery on the same day as the first surgical injury in the restenosis group, and balloon-induced injuries of the iliac arteries were performed to develop models of atherosclerosis after 4weeks (Zhou et al., 2015, 2016; Zou et al., 2020).

### Double Immunofluorescence Staining

Paraffin-embedded tissue sections were cut into slices (4μm), were deparaffinized in xylene, and were rehydrated in graded alcohol solutions. The slides were blocked antigen with 5% (v/v) goat serum for 1h. After 1× PBS wash, specimens were then stained with rabbit anti-*GRIA2* (1:100; Abcam, Cambridge, United Kingdom) and rabbit anti-*α-SMA* (1:200, Abcam, Cambridge, United Kingdom) antibodies at 4°C overnight. After rinsing with PBS for three times, immunoreactivity products were visualized by incubation with appropriate Alexa Fluor 488-conjugated secondary antibodies (1:200; Thermo Fisher Scientific, Waltham, MA, United States), 594-conjugated secondary antibodies (1:200; Thermo Fisher Scientific, Waltham, MA, United States), and DAPI (1μg/ml, Solarbio, Beijing, China) to visualize cell nuclei. After rinsing, specimens were examined with a fluorescence microscope (OLYMPUS FSX100). ImageJ software (National Institutes of Health, https://imagej.net/Citing) was used to quantify the fluorescence intensity of the cells in the intima.

### Immunohistochemical Analysis

Tissue sections deparaffinized in xylene and rehydrated with graded alcohol solutions were incubated with 1% H_2_O_2_ in methanol for 10min to block endogenous peroxidase activity. The slides were blocked antigen with 5% (v/v) goat serum for 1h, and incubated with the primary antibodies (rabbit anti-*ENPP3*, 1:100, Affinity Biosciences, United States) overnight at 4°C. After rinsing with PBS, the sections were incubated with peroxidase enzyme-conjugated goat anti-rabbit secondary antibody (#PV9001, Zhongshan, Beijing, China) for 30min at 37°C. Diaminobenzidine tetrahydrochloride (ZSBIO, Beijing, China) in PBS was used to produce a brown color. Then, the sections were counterstained with haematoxylin. ImageJ software (National Institutes of Health, https://imagej.net/Citing) was used to quantify the optical density (OD) values in the intima.

### Cell Culture and Transfection

Human vascular smooth muscle cells (HVSMCs) were purchased from Shanghai Zhong Qiao Xin Zhou Biotechnology Co., Ltd. The *GRIA2* lentiviral vector was purchased from GeneChem Corporation (Shanghai, China). HVSMCs at 30–40% confluency were transfected with Lenti-*GRIA2*-shRNA, Lenti-*GRIA2* or Lenti-NC-shRNA, according to the manufacturer’s protocol (GeneChem, Shanghai, China). After 48h of transfection, the cells were treated with 1μg/ml puromycin for 1week for the selection of stably expressing cells. The HVSMCs with stable knockdown of *GRIA2* were established. RNA was extracted and real-time (RT)-PCR experiments were conducted to confirm the efficiency of the intervention.

HVSMCs with Lenti-*GRIA2* were transiently transfected with the *ENPP3* plasmids (GeneChem, Shanghai, China) or the empty control plasmids. Briefly, Opti-MEM (Gibco-BRL/Invitrogen, Carlsbad, CA), containing 2.5μg of the *ENPP3* plasmids or the empty plasmids was mixed with 5μl of Lipofectamine 3000 transfection reagent (Thermo Fisher Scientific, Waltham, MA, United States), and the mixture was added to the cells. After 8–12h, the Opti-MEM was removed. Forty-eight hours after transfection, the transfection efficiency was evaluated by RT-PCR and western blotting.

Following the manufacturer’s instructions, the siRNAs against *ENPP3* were transfected into HVSMCs using Lipofectamine 2000 transfection reagent (Thermo Fisher Scientific, Waltham, MA, United States) after transfection with Lenti-*GRIA2*-shRNA. The siRNA knockdown efficiency was verified by RT-PCR and western blotting.

### Quantitative Real-Time Polymerase Chain Reaction

Total RNA was extracted from HVSMCs using TRIzol total RNA extraction reagent (Invitrogen, United States). cDNA was synthesized using a Light Cycler kit (Takara, Japan). *GAPDH* was used as an endogenous control for each sample. The relative expression level of the target genes was analyzed by the 2 ^− (ΔCt sample – ΔCt control)^ method. The primer sequences were as follows: *GRIA2* F: AAAGAATACCCTGGAGCACAC and R: CCAAACAATCTCCTGCATTTCC; *ENPP3* F: CGACTGCACTATGCCAAGAA and R: CATGGGCATCCTCATAGCTT; and *GAPDH* F: AGGTCGGTGTGAACGGATTTG and R: GGGGTCGTTGATGGCAACA.

### Proliferation and Migration Assays

The effect of *GRIA2* on cell proliferation was tested by the 5-ethynyl-2′ -deoxeuridine (EdU) assay kit (RiboBio Co., Ltd., Wuhan, China). First, HVSMCs were resuspended at 5×10^3^ cells/100μl and seeded into 96-well plates (Costar, Solarbio, Beijing, China) after transfection with Lenti-*GRIA2*, Lenti-*GRIA2*-shRNA, and Lenti-NC-shRNA. Then, the cells were incubated with EdU (50μm) at 37°C for 2h. Then, the VSMCs were fixed with 4% formaldehyde for 30min, incubated with glycine (2mg/ml) for 5min, and treated with 0.5% Triton X-100 for 10min. After a PBS wash, cells were incubated with 100μl of 1×Apollo reaction cocktail for 30min and treated with 0.5% Triton X-100. DNA was stained with Hoechst 33342 stain for 30min to stain all the nuclei and was then visualized with an Olympus FSX100 imaging system (Olympus, Tokyo, Japan). Five groups of confluent cells were randomly selected from each sample image. The assay was repeated at least three times.

The regulation of cell migration by *GRIA2* was verified using a 24-well Transwell (Corning-Costar, United States), and membrane insets were coated with cells. After transfection with Lenti-*GRIA2*, Lenti-*GRIA2*-shRNA, and Lenti-NC-shRNA, HVSMCs at 2×10^4^ cells/100μl were seeded in the upper chambers of Matrigel-coated 8-μm pore size Transwell filters (Corning Life Sciences, United States) while 10% serum culture medium was placed in the bottom of the plates. After incubation for 24h, the cells that migrated to the lower surface of the filter were fixed in 4% paraformaldehyde for 15min and stained with crystal violet, and the nonmigrating cells in the upper surface of the filter were removed with a cotton-tipped swab. Images of migrated cells were taken at randomly selected fields using an Olympus FSX100 imaging system (Olympus, Tokyo, Japan). Five groups of confluent cells were randomly selected from each sample image and analyzed. The assay was repeated at least three times.

### Western Blot Analysis

Protein concentrations were determined using a Bio-Rad DC Protein Assay kit (Bio-Rad Laboratories, Inc., Hercules, CA, United States). Equal amounts of protein were resolved on 10% SDS polyacrylamide gels and transferred to polyvinylidene fluoride (PVDF) membranes (EMD Millipore, Billerica, MA, United States). The membranes were subsequently incubated with rabbit anti-*GRIA2* (1:100; Abcam, Cambridge, United Kingdom), rabbit anti-*ENPP3* (1:1,000, ab190823, Abcam, Cambridge, United States), or mouse anti-*β-actin* (1:1000; A1978, Sigma Aldrich; Merck KGaA) overnight at 4°C. The next day, the membranes were incubated with the corresponding peroxidase-labeled goat anti-rabbit (1:10,000, Abcam, Cambridge, United States) or anti-mouse secondary antibodies (1:10,000, Abcam, Cambridge, United States). ImageJ software (National Institutes of Health, https://imagej.net/Citing) was used to quantify the densitometry of the bands.

### Statistical Analysis

All data were analyzed using GraphPad Prism 5 software (GraphPad Software, Inc., San Diego, CA). Student’s *t*-test was used to assess significance within two groups. One-way or two-way ANOVA analysis was used, where appropriate to assess the differences for multiple comparisons. All data are presented as the means±SEM, and the statistical significance was considered as *p*<0.05.

## Results

### Data Mining: Integrated Analysis of Two GEO Datasets Identified Significant DEGs in Atherosclerotic and Restenotic Arterial Plaques

**Figure 1:**
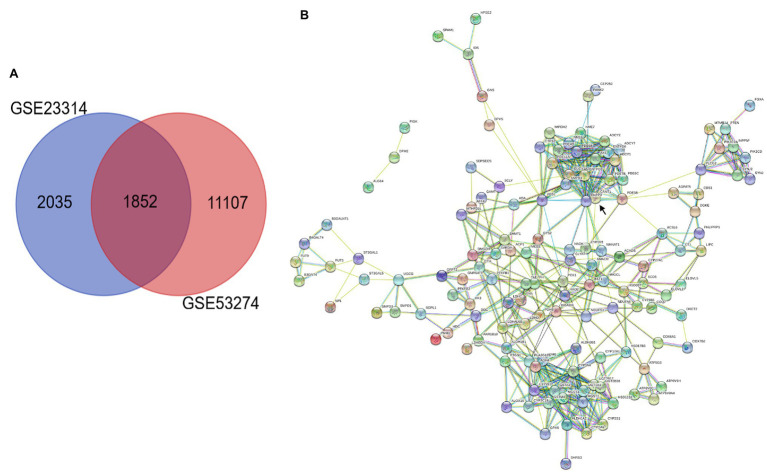
Integrated analysis of GEO datasets identified DEGs. **(A)** Venn diagrams of common DGEs between two combined datasets (GSE23314 and GSE53274). Each circle represents a dataset, and the overlap between the circles is the overlap between the datasets. **(B)** PPI network integration of the common DGEs. Each node is a gene, and the connections between nodes represent the interaction of these biological molecules. *ENPP3* (black arrow).

In this study, we performed multistep analysis to explore key DEGs and their significant biological functions in restenosis using integrated bioinformatics methods. First, we identified two GEO datasets (GSE23314 and GSE53274) that included RS tissue samples and AS tissue samples (11 RS samples and 99 AS samples). On the basis of the cut-off criteria, DEGs in each GEO dataset were identified between RS and AS. There were 4,146 DEGs in GSE23314, including 2,667 upregulated and 1,479 downregulated genes, and 12,960 DEGs in GSE53274, including 2,758 upregulated and 10,202 downregulated genes. Among these, we only found 1852 common DEGs between GSE23314 and GSE53274. The intersection plot of DEGs in these datasets is shown in Figure 1A. To characterize the functional roles of the identified DEGs, we used GO and KEGG pathway enrichment analysis. The GO analysis results showed that the DEGs were significantly enriched in the ionotropic glutamate receptor signaling pathway and synaptic transmission and glutamatergic pathways.

KEGG pathway analysis was performed using the KOBAS 3.0 system, and the DEGs were enriched in metabolic pathways. Analysis of 153 common DEGs in metabolic pathways was performed using Cytoscape, suggesting that 137 proteins interacted, among which the first 10 proteins with more connections in nodes were “*ENPP3*,” “*CYP2E1*,” “*YP2A4*,” “*ENTPD3*,” “*ADSL*,” “*PDE3B*,” “*ADCY*,” “*CANT*,” “*ADH6*,” “*ADH4*” and “*MGST1*.” *ENPP3* (black arrow) was the most important protein, connecting a total of 23 proteins, as shown in Figure 1B.

### *GRIA2* Is Highly Expressed in VSMCs in Restenotic Arteries

**Figure 2:**
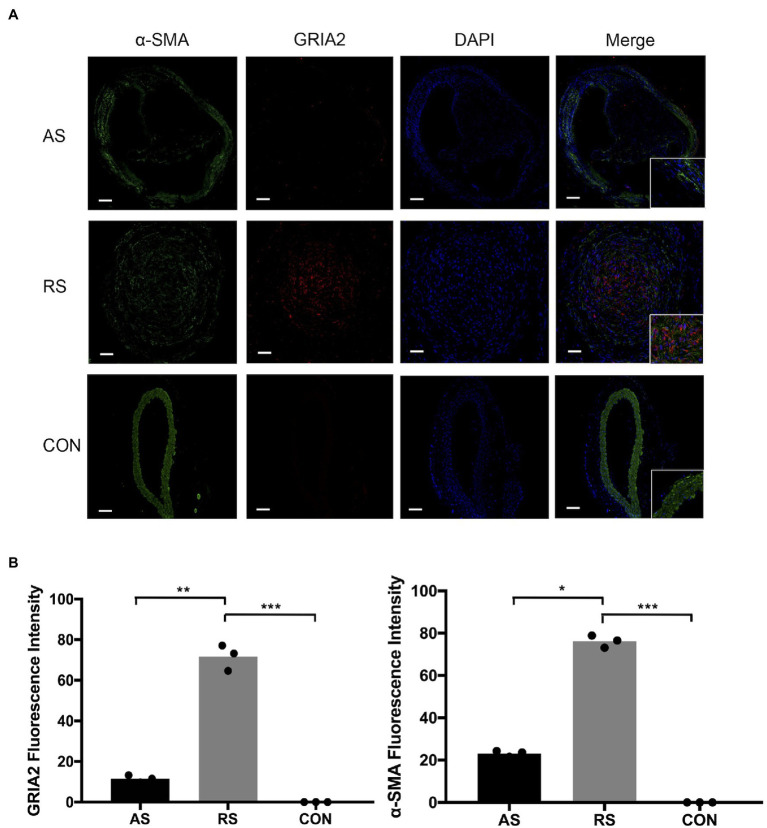
*GRIA2* is highly expressed in VSMCs in restenosis. **(A)** Double immunofluorescence staining of *GRIA2* (red) and VSMCs (green, stained with *α-SMA*) in restenotic plaque tissues, atherosclerotic plaque tissues and healthy control artery tissues is shown (*n*=3). Intensity of fluorescence is the statistical calculations in the intima. Images are representative, and **(B)** the bar graph shows the average data of three independent experiments (*n*=3). Staining was performed at 100×magnification; the right part of the picture was magnified to 200× (scale bar, 50μm). AS: atherosclerotic; RS: restenotic; CON: control; VSMCs: vascular smooth muscle cells. *Statistically significant difference (p < 0.05); **Statistically significant difference (p < 0.01); ***Statistically significant difference (p < 0.001).

Through the analysis of mRNA expression in the GEO database, we found that *GRIA2* was highly expressed in restenosis tissues. Combined with the analysis of the plaque tissues, *GRIA2* expression in restenotic plaques was significantly higher than that in atherosclerotic plaques. In addition, double immunofluorescence staining of *GRIA2* and *α-SMA* in restenosis plaque tissues revealed that the red *GRIA2* fluorescence staining in the intima was stronger than that of atherosclerotic tissues (69.2±5.3, 11.5±3.6 for RS, AS, *p*<0.01). The green *α-SMA* fluorescence staining (75.5±8.1, 22.8±5.6 for RS, AS, *p*<0.05), which is a marker of VSMCs, colocalized with the red fluorescence staining in restenotic tissues, indicating that *GRIA2* exerts its function through VSMCs. Therefore, we concluded that *GRIA2* might acts as an activator in restenosis and is associated with VSMCs behavior (Figure 2).

### *GRIA2* Is Associated With the Proliferation and Migration of VSMCs *in vitro*

**Figure 3:**
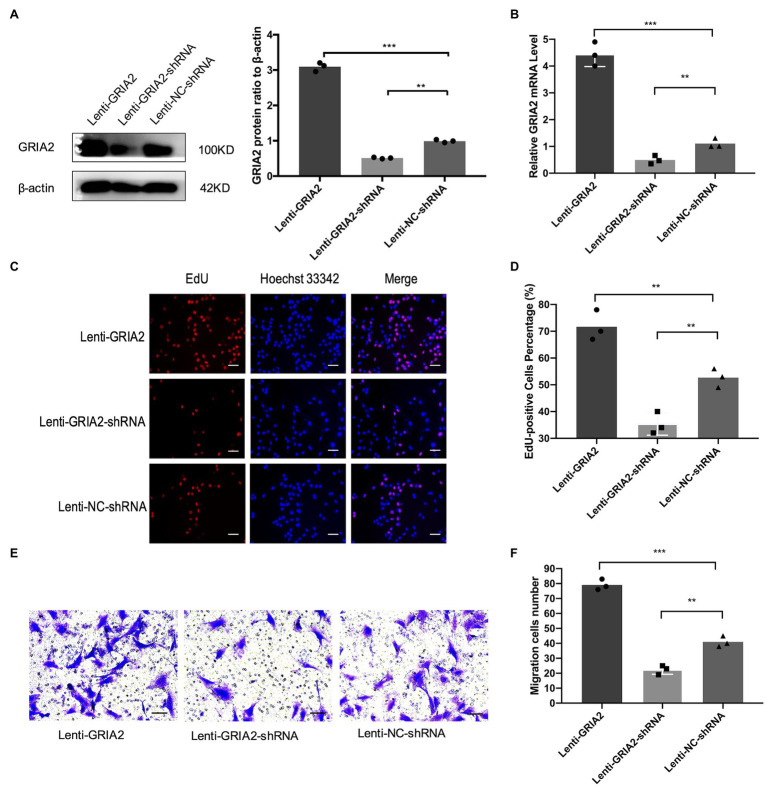
The regulation of *GRIA2* in VSMCs. **(A)** *GRIA2* protein expression was detected by western blot after cDNA and shRNA regulation of *GRIA2*. Blots are representative, and the bar graph shows the average data (*n*=3). **(B)** *GRIA2* mRNA expression was detected by real-time PCR after transfection with lentivirus carrying *GRIA2* shRNA, cDNA or empty vector (*n*=3). **(C)** EdU assay showed that increased EdU-positive VSMCs overexpressing *GRIA2* were observed (scale bar, 50μm). Images are representative, and the bar graph shows the average data of independent experiments (*n*=3). **(D)** Rate of EdU-positive VSMCs (*n*=3). **(E)** Transwell migration assay of VSMCs after upregulating *GRIA2* expression (scale bar, 100μm). Images are representative, and the bar graph shows the average data of three independent experiments (*n*=3). **(F)** Quantitative data on the migration of VSMCs (*n*=3). **Statistically significant difference (*p*<0.01) and ***statistically significant difference (*p*<0.001)

The mRNA (0.43±0.17, 4.37±0.51 for Lenti-*GRIA2*-shRNA, Lenti-*GRIA2 vs*. 1.09±0.13 for NC, *p*<0.01) and protein expression levels (0.65±0.09, 3.16±0.23 for Lenti-*GRIA2*-shRNA, Lenti-*GRIA2* vs. 1.21±0.15 for NC, *p*<0.01) of *GRIA2* significantly decreased in VSMCs transfected with Lenti-*GRIA2*-shRNA, and were upregulated in Lenti-*GRIA2* transfected VSMCs (Figures 3A,B). EdU assays were used to assess the proliferation ability of VSMCs. Figures 3C,D shows that VSMCs overexpressing *GRIA2* grew faster than the Lenti-NC-shRNA group (71.5±5.09 for Lenti-*GRIA2* vs. 50.7±3.85 for NC, *p*<0.01), and cells with *GRIA2* knockdown exhibited a slower rate of proliferation (36.8±3.83 for Lenti-*GRIA2*-shRNA vs. 50.7±3.85 for NC, *p*<0.01). Additionally, cell migration Transwell assays were conducted. Upregulation of *GRIA2* led to a marked increase in VSMCs migration (79.2±3.58 for Lenti-*GRIA2* vs. 41.3±3.82 for NC, *p*<0.001), and downregulation of *GRIA2* led to a significant decrease (22.1±2.85 for Lenti-*GRIA2*-shRNA vs. 41.3±3.82 for NC, *p*<0.01; Figures 3E,F).

### *GRIA2* Interacted With *ENPP3* in Restenosis

**Figure 4:**
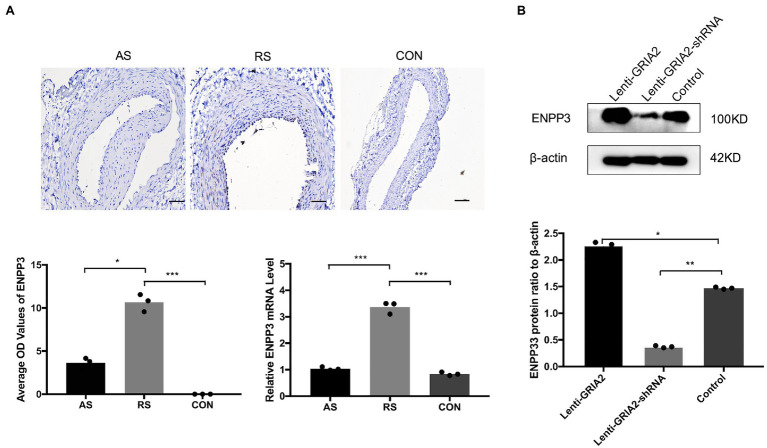
*GRIA2* interacted with *ENPP3* in restenosis. **(A)** Immunohistochemical analysis and quantitative mRNA analysis showed the expression of *ENPP3* in restenotic plaque tissues, atherosclerotic plaque tissues and healthy control artery tissues (*n*=3). Optical density (OD) values are the statistical calculations in the intima. Images are representative, and the bar graph shows the average data of three independent experiments (*n*=3). Staining was performed at 200×magnification (scale bar, 50μm). **(B)** The expression of *ENPP3* in VSMCs after upregulating *GRIA2* expression. Blots are representative, and the bar graph shows the average data (*n*=3). *Statistically significant difference (*p*<0.05), **statistically significant difference (p<0.01), and ***statistically significant difference (*p*<0.001). AS: atherosclerotic; RS: restenotic; CON: control; VSMCs: vascular smooth muscle cells.

To gain more insight into the role of *GRIA2* in restenosis, we conducted in-depth mining. KEGG enrichment analysis showed that the 1852 common DEGs in restenosis and atherosclerosis tissue samples were significantly enriched in metabolic pathways. Then, a protein–protein interaction (PPI) network was constructed. Network analysis of protein interaction data showed that ectonucleotide pyrophosphatase/phosphodiesterase 3 (*ENPP3*) acts as the central node. By reviewing the literature, we then predicted *ENPP3* as the downstream target of *GRIA2*. During *in vivo* experiments, immunohistochemical analysis results showed that *ENPP3* was significantly expressed extremely higher in restenotic plaques (10.32±1.25, 3.51±0.55 for RS, AS, *p*<0.05; Figure 4A). The analysis of mRNA expression showed that *ENPP3* was significantly higher in restenotic plaques than in atherosclerotic plaques (3.31±0.37 for RS, vs. 1.13±0.25 for AS, *p*<0.001; Figure 4A). In cell lines, the high expression of *GRIA2* in VSMCs enhanced the expression of *ENPP3* (2.29±0.36 for Lenti-*GRIA2* vs. 1.47±0.16 for Control, *p*<0.05), while downregulation of *GRIA2* downregulated *ENPP3* (0.37±0.11 for Lenti-*GRIA2*-shRNA vs. 1.47±0.16 for Control, *p*<0.01), which was consistent with the results *in vivo* (Figure 4B).

### The Promotion of VSMCs Proliferation and Migration by *GRIA2* Is Reversed by *ENPP3 in vitro*

**Figure 5:**
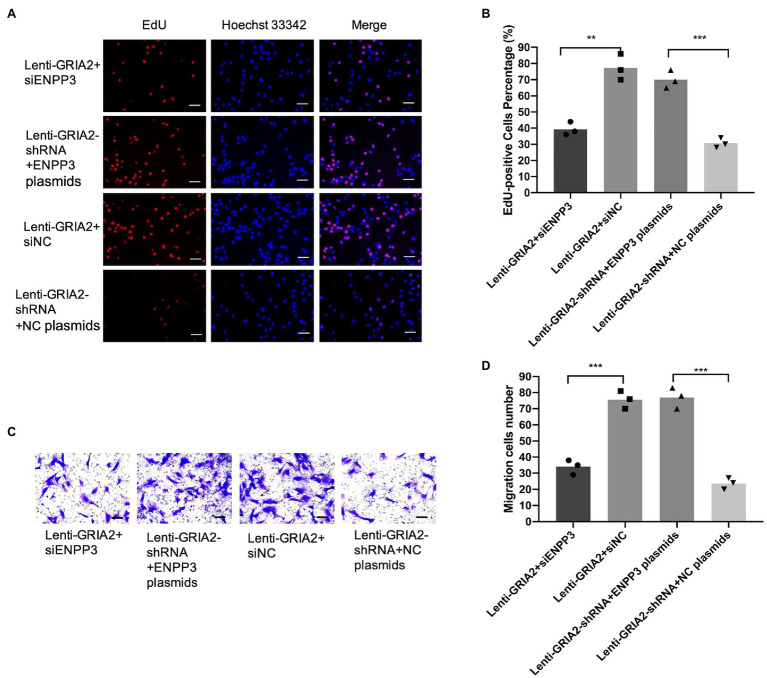
*GRIA2* interacted with *ENPP3* and regulated the proliferation and migration of VSMCs. **(A,B)** si*ENPP3* and *ENPP3* plasmids and siNC and shRNA NC plasmids were transfected into *GRIA2* lentivirus-transfected VSMCs. The EdU assay illustrated the proliferation of VSMCs (scale bar, 50μm; *n*=3). **(C,D)** Transwell assays illustrated the migration of VSMCs (scale bar, 100μm; *n*=3). Images are representative, and the bar graph shows the average data of four independent experiments (*n*=3). Images of EdU and Transwell migration assays were taken at 200× magnification. Data are presented as the mean±SEM. **Statistically significant difference (*p*<0.01) and ***statistically significant difference (*p*<0.001). VSMCs: vascular smooth muscle cells.

Based on the regulation of *ENPP3 by GRIA2*, we further investigated the effects of *GRIA2-ENPP3* on VSMCs biological functions. The results of EdU and Transwell assays showed that compared with the control group, the overexpression of *GRIA2* significantly promoted cell proliferation and migration; however, with concomitant downregulation of *ENPP3*, the promoted cell proliferation (38.9±3.88 for Lenti-*GRIA2*+si*ENPP3* vs. 76.2±5.91 for Lenti-*GRIA2*+siNC, *p*<0.01) and migration (33.2±2.69 for Lenti-*GRIA2*+si*ENPP3* vs. 73.3±4.01 for Lenti-*GRIA2*+siNC, *p*<0.001) abilities were reversed. Downregulation of *GRIA2* expression decreased VSMCs proliferation (29.2±2.63 for Lenti-*GRIA2*-shRNA+NC plasmids vs. 68.1±4.35 for Lenti-*GRIA2*-shRNA+*ENPP3* plasmids, *p*<0.001), and migration (22.5±2.39 for Lenti-*GRIA2*-shRNA+NC plasmids vs. 73.7±5.34 for Lenti-*GRIA2*-shRNA+*ENPP3* plasmids, *p*<0.001) was increased by *ENPP3* overexpression (Figures 5A–D). In summary, our experiments showed that *ENPP3* inhibits *GRIA2*-induced VSMCs.

## Discussion

Restenosis of lower extremity arteries after PTA is an unsolved conundrum. The goal of the current study was to identify candidate driver genes in restenosis through the analysis of multiple datasets of gene expression arrays. By screening, GSE23314 and GSE53274 were chosen, and through the analysis of the two datasets, we discovered significant differences in *GRIA2* expression in restenosis and atherosclerosis tissues. Moreover, *GRIA2* levels in restenosis were confirmed to be highly expressed in VSMCs in the intima. Meanwhile, in VSMCs, *GRIA2* was demonstrated to be a promoting factor of the proliferation and migration. Furthermore, we also verified the downstream target of *GRIA2*. By KEGG enrichment analysis, *ENPP3* was predicted. A series of experimental results revealed that *GRIA2* regulates *ENPP3* and modulates VSMCs proliferation and migration through *ENPP3*.

*GRIA2*, which encodes an AMPA-selective ionotropic glutamate receptor subunit (also known as *GLUR2*), affects cell membrane calcium permeability (Lee et al., 2010; Liu et al., 2010; Oakes et al., 2017; Tischbein et al., 2019; Konen et al., 2020), cell proliferation, motility, and cell death (Yoshioka et al., 1996; Takeda et al., 2000; Rzeski et al., 2001; Abdul and Hoosein, 2005; Zhang et al., 2017). *GRIA2* is normally expressed in the central nervous system under physiological conditions (Song et al., 1998; Liu and Cull-Candy, 2000; Nakagawa et al., 2005), but *GRIA2* can be detected in various oncogenic conditions through unknown mechanisms. Abnormal *GRIA2* expression has also been demonstrated in several tumors and tumor cell lines (Yoshioka et al., 1996; Takeda et al., 2000; Rzeski et al., 2001; Abdul and Hoosein, 2005; Ishiuchi et al., 2007; Ni et al., 2020) and is thought to mediate increased cell proliferation.

*GRIA2* expression has not yet been characterized in restenosis. We discovered, for the first time, that *GRIA2* accumulated in restenotic plaques and may participate in the restenosis process. VSMCs were the dominant components of restenotic plaques. The migration and proliferation of VSMCs are critical events in the development of restenosis (Bennett et al., 2016). We verified the function of *GRIA2* in VSMCs. The results showed that overexpression of *GRIA2* significantly promoted the proliferation and migration of VSMCs, and downregulation of *GRIA2* inhibited VSMCs behavior, indicating that *GRIA2* is a regulator of restenosis. Glutamate receptor 2, encoded by *GRIA2*, is an important subunit of the AMPA-selective glutamate receptor. Glutamate receptor 2 incorporation is known to greatly reduce the permeability of receptors to Ca^2+^ and plays an important role in cellular Ca^2+^ homeostasis (Lee et al., 2010; Liu et al., 2010; Oakes et al., 2017; Tischbein et al., 2019; Konen et al., 2020). Recent evidence has shown that *GRIA2* expression levels are linked with VSMC phenotypic switching. Under physiological conditions, VSMCs normally maintain a contractile phenotype. In response to vascular injury, VSMCs undergo phenotypic switching to a dedifferentiated state characterized by excessive proliferation and migration. In addition, studies have also reported that *GRIA2* signaling regulates the expression of a gene set involved in the MAPK/ERK signaling pathway (Gallina et al., 2021). Because the MAPK/ERK pathway plays a crucial role in cell proliferation, differentiation, and migration, the mechanism by which *GRIA2* regulates VSMCs proliferation and migration may be through the MAPK/ERK signaling pathway.

To gain more insight into the mechanism of *GRIA2* in restenosis, we predicted the downstream target of *GRIA2* through the reanalysis of the GSE23314 and GSE53274 datasets. A total of 1852 common DEGs were subjected to KEGG enrichment analysis. The results showed that the DEGs were highly enriched in metabolic pathways. Data on PPIs provide valuable insight into molecular networks. By PPI network analysis, *ENPP3*, an N-glycosylated protein (Boggavarapu et al., 2016), was predicted to be the core protein in restenosis. *ENPP3* is a newly described molecule and, therefore, is less studied. The expression of *ENPP3* in general has mainly been reported in basophil and mast cells (Buhring et al., 2004; Trapero et al., 2018). Recent studies have shown that *ENPP3* is involved in the invasion of tumor cells (Yano et al., 2003, 2004), which is similar to the process of VSMCs migration and proliferation from the media to the intima and forms restenotic plaques. Here, in our study, the expression and localization of *ENPP3* were detected. In restenotic plaques, the *ENPP3* was also increased and located in VSMCs in the intima, correlated with *GRIA2* expression, suggesting that *ENPP3* might interact with *GRIA2*. *ENPP3* is a type II transmembrane protein (Buhring et al., 2001) that is involved in hydrolysis of extracellular nucleotides and possesses ATPase and ATP pyrophosphatase activities (Reigada et al., 2005; Levesque et al., 2007). No previous report has demonstrated the expression and interaction between *GRIA2* and *ENPP3*. However, studies have shown that *ENPP3* is usually located in neurons (Nishida et al., 2018). Moreover, as *ENPP3* is a membrane protein, and *GRIA2* possesses a certain degree of cell membrane protein modulation. However, these results indicate that GRIA2 promotes VSMCs effects through *ENPP3*. To investigate the effect of *GRIA2* on *ENPP3*, we modulated *GRIA2*. With *GRIA2* intervention, the expression of *ENPP3* was positively regulated. Our results further showed that *GRIA2*-promoted VSMCs proliferation and migration were inhibited by ENPP3 repression, implying that *GRIA2* interacted with *ENPP3* and further regulated VSMCs behavior in restenosis. Further studies are needed to clarify their potential interactions. In present study, we used a double-balloon injury animal model, which consisted of two steps injuries: the first balloon injury was to induce atherosclerosis plaques and, after the plaques were established, the second balloon injury was performed in the same place to mimic the condition of PTA. We found the plaques developed after the two steps injuries are typically hyperplasia foci of VSMCs and extracellular matrix, which is more closely to human restenosis pathology than simple balloon injury protocol (Strauss et al., 1996; Zhou et al., 2016; Zou et al., 2020).

Our results have demonstrated that *GRIA2* has a significant impact on restenosis by modulating VSMCs proliferation and migration. We also explored the mechanism by which upregulated *GRIA2* regulates VSMCs behavior as the upstream of *ENPP3*. Targeting *GRIA2-ENPP3* may have potential in the treatment of restenosis post-PTA in lower extremity arteries.

## Data Availability Statement

The datasets presented in this study can be found in online repositories. The names of the repository/repositories and accession number(s) can be found in the article/supplementary material.

## Ethics Statement

The animal study was reviewed and approved by the Ethics Committee of Xuanwu Hospital affiliated with Capital Medical University.

## Author Contributions

MZ and YG designed the methods and analyzed the data and results. MZ and LQ wrote the manuscript and prepared figures. All authors contributed to the article and approved the submitted version.

## Conflict of Interest

The authors declare that the research was conducted in the absence of any commercial or financial relationships that could be constructed as a potential conflict of interest.

## Publisher’s Note

All claims expressed in this article are solely those of the authors and do not necessarily represent those of their affiliated organizations, or those of the publisher, the editors and the reviewers. Any product that may be evaluated in this article, or claim that may be made by its manufacturer, is not guaranteed or endorsed by the publisher.

## Footnotes

^1^ www.ncbi.nlm.nih.gov/geo/geo2r

^2^ https://david.ncifcrf.gov/

^3^ http://kobas.cbi.pku.edu.cn/

## Abbreviations

PTA: Percutaneous transluminal angioplasty
PAD: Peripheral artery disease
DEGs: Differentially expressed genes
GEO: Gene Expression Omnibus
GO: Gene Ontology
KEGG: Kyoto Encyclopedia of Genes and Genomes
VSMCs: Vascular smooth muscle cells
HVSMCs: Human vascular smooth muscle cells
DCBs: Drug-coated balloons
*GRIA2*: Glutamate ionotropic receptor AMPA type subunit 2
SD: Sprague–Dawley
qRT-PCR: Quantitative real-time polymerase chain reaction
EdU: 5-Ethynyl-2′-deoxeuridine
AS: Atherosclerotic
RS: Restenotic
PPI: Protein–protein interaction
*ENPP3*: Ectonucleotide pyrophosphatase/phosphodiesterase 3
PVDF: Polyvinylidene fluoride
